# Triglyceride–glucose index and preserved ratio impaired spirometry in 89,809 individuals without self-reported overt clinical disease

**DOI:** 10.3389/fmed.2026.1880988

**Published:** 2026-09-25

**Authors:** Hyun-Il Gil, Sungwon Lim, Si-Young Lim, Soo-Youn Ham, Jonghoo Lee, Jae-Uk Song

**Affiliations:** 1 Division of Pulmonary and Critical Care Medicine, Department of Internal Medicine, Kangbuk Samsung Hospital, Sungkyunkwan University School of Medicine, Seoul, Republic of Korea; 2 Department of Nursing, Dongguk University-WISE, Gyeongju, Republic of Korea; 3 Department of Radiology, Kangbuk Samsung Hospital, Sungkyunkwan University School of Medicine, Seoul, Republic of Korea; 4 Department of Internal Medicine, Jeju National University Hospital, Jeju National University School of Medicine, Jeju, Republic of Korea

**Keywords:** Asian population, insulin resistance, preserved ratio impaired spirometry, restrictive pattern, spirometry, triglyceride-glucose index

## Abstract

**Introduction:**

While both preserved ratio impaired spirometry (PRISm) and triglyceride glucose index (TyG) are established risk factors for cardiometabolic diseases, the metabolic characteristics and prevalence of PRISm differ by ethnic group. However, this association has not been directly examined in Asian populations. Therefore, we aimed to investigate whether TyG is associated with PRISm in a large Korean population without self-reported overt clinical disease.

**Methods:**

In this cross-sectional study, 89,809 adults (46,739 men; mean age: 38.5 years) with health examinations in 2019 were divided into quartiles according to their TyG. Lung function phenotypes were evaluated using fixed cut-offs and lower limit of normal (LLN) criteria, with LLN defined as the 5th percentile of our study population. Adjusted odds ratios (aORs) and 95% confidence intervals (CIs) for PRISm were estimated and compared to the lowest TyG quartile (reference) group.

**Results:**

The mean TyG was 8.34 ± 0.57, and the prevalence of PRISm was 3.4%, showing a significant increase across TyG quartiles (*p* for trend < 0.001). Compared with the lowest quartile, the aORs (95% CI) for PRISm were 1.157 (1.030–1.299), 1.232 (1.085–1.398), and 1.368 (1.193–1.567) 1 across the increasing quartiles of TyG (*p* for trend < 0.001). A similar increasing trend was observed for the spirometric restrictive pattern (SRP) across TyG quartiles (*p* for trend < 0.001), whereas no significant trend was found for the obstructive pattern (OP) (*p* = 0.135). Results obtained using fixed cut-off criteria were highly consistent with those based on LLN criteria.

**Discussion:**

In conclusion, higher TyG quartiles were associated with PRISm in middle-aged Koreans without self-reported overt clinical disease, suggesting the potential link between metabolic status and PRISm risk. However, longitudinal studies are required to confirm these findings.

## 1 Introduction

Chronic obstructive pulmonary disease (COPD) and cardiovascular metabolic diseases (CVMDs) are highly prevalent conditions that impose substantial medical and financial burdens on society and contribute significantly to global morbidity and mortality (1). Both conditions frequently coexist (2, 3). Hence, the early identification of modifiable conditions that precede both COPD and CVMDs, along with timely intervention in their early stages, may help delay disease progression and reduce the burden on the healthcare system, given their largely irreversible nature.

PRISm is defined by a forced expiratory volume in one second (FEV_1_)/forced vital capacity (FVC) ratio of 0.7 or more, with an FEV_1_ value below 80% of the predicted value on spirometry (3, 4). PRISm is increasingly recognized among middle-aged and older adults, with a pooled prevalence of 12%, although its estimates vary widely (4.5%–45.7%) depending on the definition, reference values, and study population (4, 5). PRISm is linked to various CVMDs and COPD as well as increased all-cause and cardiorespiratory mortality in the general population (3–7). It is also a dynamic condition, with individuals showing variable longitudinal transitions to normal or obstructive patterns (OP), while some remain stable (3, 4). In this context, PRISm represents a precursory clinical phenotype of lung dysfunction related to COPD (3, 4) and CVMDs (7). Despite its clinical importance and global prevalence, PRISm has historically been neglected (3–5). Consequently, there are currently no established diagnostic or management guidelines for PRISm (4), and spirometry is rarely performed in asymptomatic individuals in routine care or screening settings. Therefore, early identification of PRISm-related risk factors and timely intervention may help reduce the morbidity and mortality associated with CVMDs and COPD-related complications.

Interestingly, insulin resistance (IR) plays a key role in various respiratory conditions, including COPD (2, 8, 9) and CVMDs (9–12), and is associated with a decline in FEV_1_ and FVC (13). These findings suggest a potential link between IR and PRISm, and indicate that IR may be a modifiable risk factor for PRISm, with implications for reducing PRISm-related morbidity and mortality. In public health screening and large-scale epidemiological studies, TyG has been increasingly used as an IR surrogate marker because of its convenience and strong predictive performance (14). Moreover, TyG is closely associated with COPD (15), CVMDs (14) and impaired lung function (13).

However, studies on these associations are limited. Although one study demonstrated a positive association between TyG and PRISm in the US population (16), its primary focus was on dietary inflammation and PRISm, with TyG serving as a mediator. Furthermore, metabolic characteristics (17, 18) and prevalence of PRISm (5) differ by race and ethnicity. Consequently, the relationship remains unclear, particularly in Asian populations. Therefore, this study aimed to investigate the association between TyG and PRISm in a large sample of Korean participants without comorbidities.

## 2 Materials and methods

### 2.1 Study design and population

This cross-sectional study is an extension analysis utilizing a subset of data from our previously published cohort research, employing comparable baseline measurement method (13). While our prior work demonstrated the close association between higher TyG values and decreased FEV1 and FVC, the present study specifically focuses on PRISm. Although a few Western studies have evaluated the TyG in relation to PRISm, their findings remain inconsistent (16, 19, 20). Moreover, given the substantial ethnic variations in metabolic characteristics (17, 18) and PRISm prevalence (4, 5), findings from Western cohorts may not be directly generalizable to Asian populations who exhibit distinct metabolic profiles (17, 18). Currently, data evaluating the TyG–PRISm association in Asians are lacking. Therefore, this study extends our previous findings by addressing these literature inconsistencies and establishing the independent relationship between TyG and PRISm in an Asian population.

The study population was drawn from 214,551 individuals aged 18 years or older who underwent a comprehensive health examination at Kangbuk Samsung Hospital Health Screening Center in 2019. Under the South Korean Industrial Safety and Health Law, employees are required to undergo annual or biennial health examinations, which are free of charge. Most participants were employees or had family members from various companies or local governmental organizations, whereas the remainder were voluntarily enrolled in the screening program.

From the initial cohort, we extracted data from 213,477 participants with recorded TyG and spirometric data. After further exclusion of 123,668 participants without lifestyle or medical information and with a self-reported history or treatment of a medical condition, 89,809 participants were included in the final analysis (Figure 1). However, the questionnaire collected medical history through binary (yes/no) items, which precluded retrieval of granular comorbidity information. Therefore, some individuals met more than one exclusion criterion.

**Figure 1:**
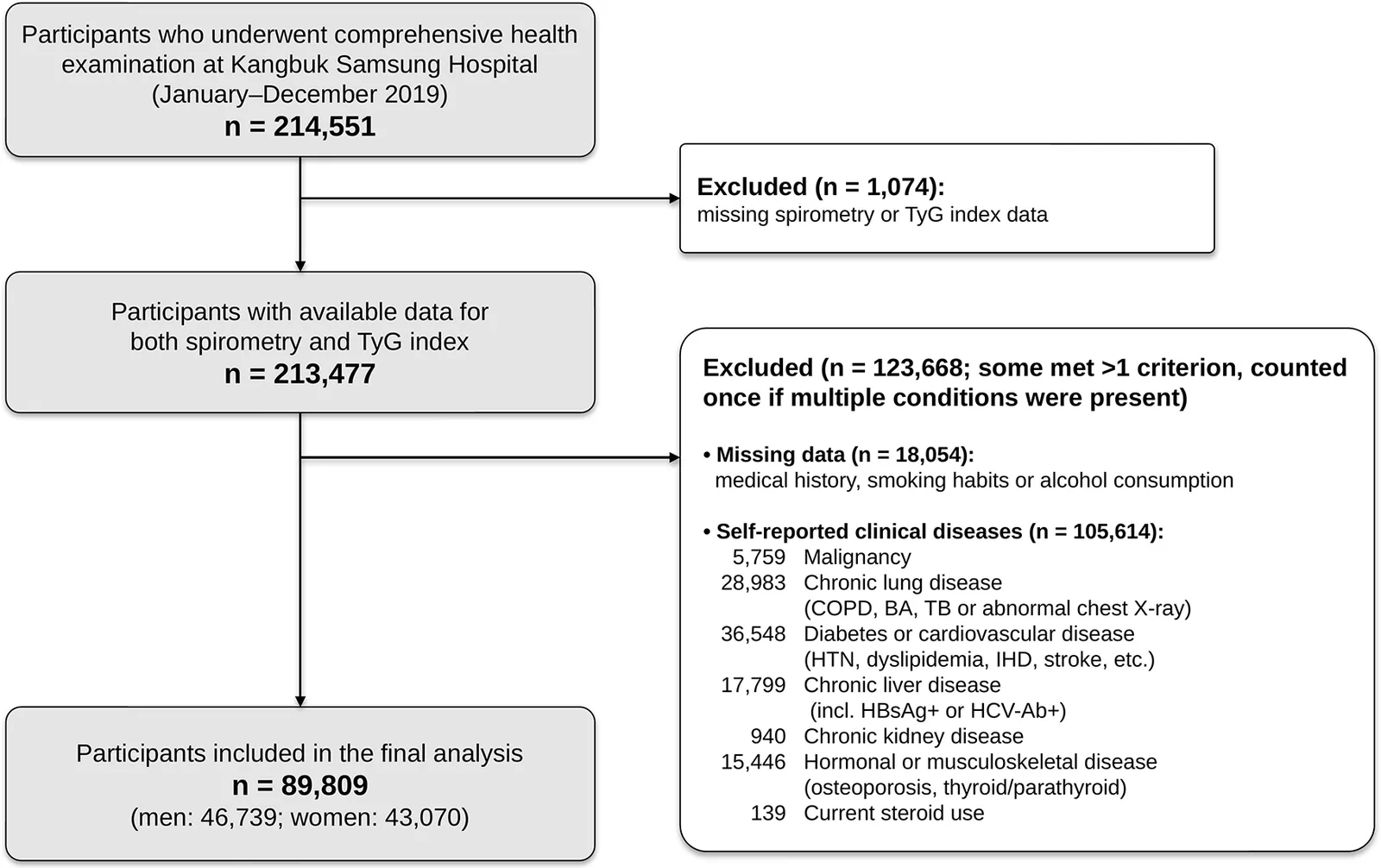
Flow chart of the study participants. BA, bronchial asthma; COPD, chronic obstructive pulmonary disease; HBsAg, hepatitis B virus surface antigen; HCV-Ab, hepatitis C virus antibody; HTN, hypertension; IHD, ischemic heart disease; TB, tuberculosis; TyG, triglyceride-glucose index.

This study was approved by the Institutional Review Board of Kangbuk Samsung Hospital (KBSMC 2024-01-038) and conducted in accordance with the 1975 Declaration of Helsinki and its later amendments. This study adhered to the Strengthening the Reporting of Observational Studies in Epidemiology (STROBE) reporting guidelines. The requirement for informed consent was waived due to the use of de-identified data in our retrospective analyses.

### 2.2 Data collection, anthropometric measurements, and laboratory tests

The comprehensive health screening program collected information on sociodemographic characteristics, lifestyle behaviors, anthropometric measurements, and laboratory parameters. Data on demographics, smoking and drinking habits, physical activity, medical history, medication use, and current symptoms were obtained using standardized self-administered questionnaires. Smoking status was classified as nonsmoker, ex-smoker, or current smoker. Alcohol intake was quantified from the frequency and usual amount of alcohol consumed per drinking day and then classified as none, non-heavy (<20 g ethanol/day), or heavy (≥20 g ethanol/day). Physical activity was self-reported and quantified as the number of days per week with at least 30 min of moderate-intensity exercise sufficient to induce slight breathlessness. Participants who met this threshold on three or more days per week were classified as regular exercisers (21).

Trained nurses obtained the anthropometric data and collected blood samples following the standardized protocols described in our previous publications (13, 21). Height and weight were measured using automated instruments (InBody 3.0, Inbody 720, Biospace Co., Seoul, Republic of Korea) with participants wearing hospital gowns without their shoes. Waist circumference (WC) was measured at the midline between the lower rib margin and the iliac crest. The appropriate WC cut-off for central obesity in Koreans was defined as 90 cm in men and 85 cm in women (22). Body mass index (BMI) was calculated by dividing the weight (kg) by the square of the height (m^2^). Obesity was defined as a body mass index (BMI) ≥ 25 kg/m^2^ (13, 21). Blood pressure (BP) was measured using a standard sphygmomanometer after at least 5 min of seated rest. Two measurements were taken at 5-min intervals and averaged for the analysis. Mean BP (MBP) was calculated as diastolic BP + 0.33 × (systolic BP − diastolic BP).

Blood samples were collected after fasting for at least 10 h. Methods for assessing liver enzymes, creatinine, lipid profiles, fasting blood glucose (FBS) levels, and glycated hemoglobin (HbA1c) levels have been previously reported (13, 21). All assays showed excellent reproducibility, with total coefficients of variation <5% for both normal-range and high-level quality control materials during the study period. Triglycerides (TG) were measured using an enzymatic colorimetric assay on a Cobas 8000 c702 chemistry analyzer (Roche Diagnostics, Tokyo, Japan). FBS levels were determined using the hexokinase method on a Cobas Integra 800 apparatus (Roche Diagnostics). Total coefficients of variation for TG and FBS were 0.94%–1.36% and 0.65%–1.63% for the low-level quality control materials and 0.90%–1.16% and 0.55%–1.08% for the high-level quality control materials, respectively. The TyG was calculated as the natural logarithm of [TG (mg/dL) × FBS (mg/dL)/2] (14). Metabolic syndrome (MS) was defined using the National Cholesterol Education Program Adult Treatment Panel III (NCEP-ATPIII) (23). All laboratory analyses were performed at the Laboratory Medicine Department of the Kangbuk Samsung Hospital, which is accredited and participates annually in external quality assessment programs conducted by the Korean Association of Quality Assurance for Clinical Laboratories.

### 2.3 Lung function measurements

Spirometry was performed according to the American Thoracic Society/European Respiratory Society guidelines (24), using the Vmax22 system (Sensor-Medics, Yorba Linda, CA, USA). Forced expiratory volume in one second (FEV1) and forced vital capacity. (FVC) were obtained in the pre-bronchodilator state. Only tests that met both ATS/ERS acceptability and repeatability criteria (obtaining the highest values from three or more acceptable maneuvers) were included in the analysis.

The reference values for FEV1 and FVC were estimated using prediction equations derived from a representative Korean population sample (25). The percent predicted values for FEV_1_ (FEV_1_%) and FVC (FVC%) were computed by dividing the measured values (L) by the corresponding predicted values and expressing the results as percentages. The FEV_1_/FVC ratio was calculated directly from FEV_1_ and FVC measurements.

In our cohort, the fifth percentile of the spirometric values was used as the LLN, which is consistent with the method adopted in a prior study (26). PRISm was defined as a preserved FEV_1_/FVC ratio (≥LLN) with a reduced FEV_1_ (<LLN) (3, 4). Because total lung capacity (TLC) was not routinely measured, spirometry alone cannot definitively confirm true restrictive ventilatory defect (24); thus, we intentionally used the term spirometric restrictive pattern (SRP) rather than restrictive pattern to avoid diagnostic overstatement. SRP was defined as FEV_1_/FVC ≥ LLN and FVC < LLN, whereas an obstructive pattern (OP) was defined as FEV_1_/FVC < LLN (24). Although using internally derived 5th percentile thresholds (LLN) helps minimize systematic misclassification of impaired lung function introduced by external reference equations derived from Western or disparate populations, when applying external reference equations derived from Western or disparate populations, these cutoffs could theoretically shift the baseline diagnostic threshold due to the diverse metabolic profiles of our cohort. Therefore, we additionally applied conventional fixed criteria (FEV1/FVC ≥ 0.7 and FEV1% < 80 for PRISm; FEV1/FVC ≥ 0.7 and FVC% < 80 for SRP and FEV1/FVC < 0.7 for OP) based on validated Korean reference equations (25), to test the robustness of our main findings.

### 2.4 statistical analyses

The demographic and clinical characteristics of the study participants were compared across the TyG quartiles. Continuous data are presented as the mean ± standard deviation (SD) when normally distributed, or as the median with interquartile range (IQR) otherwise; categorical data are summarized as frequencies and percentages. The Kolmogorov–Smirnov test was used to assess the normality of continuous variables. Because our study used pre-existing health-screening data, detailed records of the reasons for missing values were not available. Little's missing completely at random (MCAR) test was performed to assess the pattern of missingness. The test results were not significant (*p* = 0.0827), indicating that the missing data are consistent with the MCAR mechanism. Differences among TyG quartiles were analyzed using the chi-square test or Fisher's exact test for categorical variables and one-way ANOVA or the Kruskal–Wallis test for continuous variables.

For further categorical analyses, all covariates were dichotomized into high/low or presence/absence categories, and differences across the four TyG quartile groups were tested using the chi-square test or Fisher's exact test. To examine the independent association between TyG quartiles and impaired lung function patterns, multivariable logistic regression analyses were conducted with sequential adjustment models. Model 1 was adjusted for age, sex, smoking and alcohol habits, regular exercise and MBP. Models 2 through 5 separately evaluated individual metabolic and glycemic parameters (Model 2: Model 1 + WC; Model 3: Model 1 + MS; Model 4: Model 1 + lipid profile such as total cholesterol (TC), high-density lipoprotein (HDL) cholesterol, and low-density lipoprotein (LDL) cholesterol; Model 5: Model 1 + HbA1c). To prevent severe multicollinearity and overadjustment, Model 6 was constructed as a comprehensive model adjusting for baseline demographic factors, lifestyle covariates, MS, and non-redundant, non-collinear metabolic and organ function markers with a variance inflation factor (VIF < 2.0). Individual components encompassed within MS, such as MBP, WC, and HDL cholesterol, were excluded from Model 6. Given the partial overlap and non-interchangeability between PRISm and spirometric restrictive pattern (SRP), we conducted additional subgroup analyses across mutually exclusive outcome categories (PRISm only, PRISm with SRP, and pure SRP) to evaluate their respective associations with TyG. To enhance the robustness of our findings, we also performed subgroup analyses stratified by sex, age group, BMI or obesity status, smoking status, and MS status, along with tests for interaction. Two-sided *p* values below 0.05 indicated statistical significance. All analyses were carried out in SPSS Statistics version 24.0 (IBM Corp., Armonk, NY, USA).

## 3 Results

### 3.1 Baseline characteristics of the study population

Table 1 shows the baseline characteristics of the 89,809 eligible participants (52.0% men; 38.5 ± 7.3 years) according to TyG quartiles. Mean BMI and TyG were 23.2 ± 3.3 kg/m^2^ and 8.34 ± 0.57, respectively, and 18.2% of participants had MS. With increasing TyG quartiles, age, male sex, BMI, WC, smoking, alcohol intake, ALT, creatinine, and conventional cardiometabolic parameters (FBS, HbA1c, TC, TG, LDL-cholesterol, BP, and MS) increased, whereas HDL cholesterol decreased (all *p* < 0.001). Spirometric indices, including FEV_1_, FEV_1_%, FVC, FVC%, and FEV_1_/FVC ratio, also decreased progressively across higher TyG quartiles (*p* < 0.001).

**Table 1:** Baseline characteristics of study participants by quartiles of TyG.

Characteristics	Overall subjects (*N* = 89,809)	Q1 (≤7.97) (*N* = 22,572)	Q2 (7.98–8.33) (*N* = 22,784)	Q3 (8.34–8.73) (*N* = 21,874)	Q4 (≥8.74) (*N* = 22,579)	*p* value
Age (years)	38.5 ± 7.3	36.7 ± 7.2	38.0 ± 7.2	39.2 ± 7.3	40.3 ± 7.1	<0.001
Sex (male)	46,739 (52.0)	6,141 (27.2)	9,314 (40.9)	13,122 (60.0)	18,162 (80.4)	<0.001
Waist circumference (cm) (*N* = 89,804)	80.2 ± 9.5	74.4 ± 7.6	77.7 ± 8.4	81.8 ± 8.6	87.0 ± 8.3	<0.001
BMI (kg/m^2^)	23.2 ± 3.3	21.5 ± 2.5	22.4 ± 2.9	23.7 ± 3.1	25.4 ± 3.2	<0.001
Smoking (pack-years)	3.13 ± 6.50	1.13 ± 3.80	1.94 ± 5.07	3.44 ± 6.70	6.04 ± 8.39	<0.001
Alcohol consumption (g/day)	12.98 ± 20.46	9.03 ± 15.52	10.60 ± 17.34	13.41 ± 20.47	18.91 ± 25.67	<0.001
Moderate physical activity frequency (times/week) (*N* = 89,544)	0.85 ± 1.41	0.93 ± 1.52	0.85 ± 1.42^a^	0.84 ± 1.38^a^	0.78 ± 1.31	<0.001
Total bilirubin (mg/dL) (*N* = 89,794)	0.79 ± 0.36	0.79 ± 0.37^a^^,^^b^	0.78 ± 0.36	0.79 ± 0.36^a^^,^^c^	0.79 ± 0.34^b^^,^^c^	<0.001
ALT (U/L) (*N* = 89,648)	17 (12–25)	14 (11–18)	15 (11–20)	18 (13–25)	25 (18–37)	<0.001
Serum creatinine (mg/dL)	0.80 ± 0.17	0.74 ± 0.16	0.77 ± 0.17	0.82 ± 0.17	0.87 ± 0.16	<0.001
Total cholesterol (mg/dL)	192 ± 31	179 ± 28	187 ± 28	196 ± 29	207 ± 31	<0.001
Triglyceride (mg/dL)	89 (64–130)	53 (45–59)	76 (70–83)	106 (97–117)	173 (147–219)	<0.001
HDL cholesterol (mg/dL) (*N* = 89,807)	62 ± 17	73 ± 15	67 ± 15	59 ± 14	50 ± 12	<0.001
LDL cholesterol (mg/dL) (*N* = 89,806)	123 ± 30	107 ± 26	118 ± 27	130 ± 29	140 ± 30	<0.001
Fasting glucose (mg/dL)	93.4 ± 10.7	87.5 ± 7.1	91.7 ± 6.7	94.5 ± 7.4	99.9 ± 14.9	<0.001
HbA1c (%) (*N* = 89,805)	5.4 ± 0.4	5.3 ± 0.3	5.4 ± 0.3	5.4 ± 0.3	5.6 ± 0.5	<0.001
Systolic BP (mmHg)	108 ± 12	103 ± 10	106 ± 11	109 ± 11	115 ± 12	<0.001
Diastolic BP (mmHg)	70 ± 9	66 ± 8	68 ± 8	71 ± 9	75 ± 9	<0.001
MBP (mmHg)	83 ± 10	78 ± 8	81 ± 9	84 ± 9	88 ± 10	<0.001
Metabolic syndrome	16,369 (18.2)	210 (0.9)	821 (3.6)	2,275 (10.4)	13,063 (57.9)	<0.001
TyG i	8.34 ± 0.57	7.70 ± 0.22	8.15 ± 0.10	8.52 ± 0.11	9.13 ± 0.35	<0.001
Measured FEV1 (L)	3.30 ± 0.68	3.51 ± 0.63	3.37 ± 0.69	3.21 ± 0.69	3.10 ± 0.64	<0.001
FEV1%	98.00 ± 10.71	99.27 ± 10.81	98.71 ± 10.77	97.75 ± 10.58	96.27 ± 10.44	<0.001
Measured FVC (L)	4.00 ± 0.87	4.34 ± 0.79	4.11 ± 0.87	3.87 ± 0.86	3.67 ± 0.80	<0.001
FVC%	98.31 ± 10.69	99.26 ± 10.89	98.78 ± 10.75	98.29 ± 10.59	96.89 ± 10.37	<0.001
FEV1(L)/FVC(L) ratio	0.829 ± 0.059	0.847 ± 0.061	0.834 ± 0.059	0.822 ± 0.056	0.812 ± 0.053	<0.001

Data are presented as means ± standard deviation, median (interquartile range), or number of subjects with percentages in parentheses.

We recorded the number of participants for whom clinical parameters were available. Unless otherwise indicated, data from 89,809 participants were available.

Detailed documentation of missing values for some variables was unavailable because our study utilized pre-existing health check-up data. However, the MCAR test result was not significant (*p* = 0.0827), indicating that the data were completely missing at random.

ALT, alanine aminotransferase; BMI, body mass index; BP, blood pressure; FEV1%, percent predicted forced expiratory volume in 1 s; FVC%, percent predicted forced vital capacity; HbA1c, hemoglobin A1c; HDL, high-density lipoprotein; LDL, low-density lipoprotein; MBP, mean blood pressure; TyG, triglyceride-glucose index.

MBP = diastolic BP + 0.33 × (average systolic BP − average diastolic BP).

a,b,c The same letters denote no significant differences between the designated groups in *post-hoc* analyses. Otherwise, *post hoc* analyses (Bonferroni correction) showed significant differences between the groups.

### 3.2 Comparison of clinical and laboratory parameters among groups stratified by quartile of TyG

Table 2 presents a comparison of the clinical and laboratory characteristics among the four groups divided by TyG quartile. Participants in the higher TyG quartiles were more likely to be men, older, have a higher BMI and WC, and engage in smoking and alcohol consumption (all *p* < 0.001). Furthermore, adverse cardiometabolic parameters such as lipid profiles, FBS, HbA1c, MBP, and MS became more common with increasing quartiles of TyG (all *p* < 0.001), whereas total bilirubin showed a decreasing trend. The higher TyG groups also showed a greater proportion of elevated ALT and creatinine levels (all *p* < 0.001).

**Table 2:** Comparisons of demographic and clinical parameters between four groups stratified by quartile of TyG.

Demographics and clinical parameters	All subjects (*N* = 89,809)	Q1 (≤7.97) (*N* = 22,572)	Q2 (7.98–8.33) (*N* = 22,784)	Q3 (8.34–8.73) (*N* = 21,874)	Q4 (≥8.74) (*N* = 22,579)	*p* value
Age (≥39 years)	39,361 (43.8)	7,498 (33.2)	9,349 (41.0)	10,387 (47.5)	12,127 (53.7)	<0.001
Sex (male)	46,739 (52.0)	6,141 (27.2)	9,314 (40.9)	13,122 (60.0)	18,162 (80.4)	<0.001
High waist circumference (*N* = 89,804)^a^	19,967 (22.2)	2,098 (9.3)	3,538 (15.5)	5,450 (24.9)	8,881 (39.3)	<0.001
BMI (≥25 kg/m^2^ = obesity)	23,981 (26.7)	1,998 (8.9)	3,937 (17.3)	6,464 (29.6)	11,582 (51.3)	<0.001
Current smokers	13,775 (15.3)	1,427 (6.3)	2,365 (10.4)	3,638 (16.6)	6,345 (28.1)	<0.001
Heavy alcohol intake (>20 g/day)	16,429 (18.3)	2,399 (10.6)	3,143 (13.8)	4,227 (19.3)	6,660 (29.5)	<0.001
Regular exercise (≥3 times/week) (*N* = 89,544)	11,805 (13.2)	3,497 (15.5)	3,056 (13.5)	2,786 (12.8)	2,466 (11.0)	<0.001
Elevated bilirubin (>1.9 mg/dL) (*N* = 89,794)	1,192 (1.3)	370 (1.6)	301 (1.3)	267 (1.2)	254 (1.1)	<0.001
Elevated ALT (>40 U/L) (*N* = 89,648)	7,434 (8.3)	491 (2.2)	801 (3.5)	1,699 (7.8)	4,443 (19.7)	<0.001
Elevated serum creatinine (>1.2 mg/dL)	209 (0.2)	24 (0.1)	43 (0.2)	61 (0.3)	81 (0.4)	<0.001
Hypercholesterolemia (≥220 mg/dL)	15,296 (17.0)	1,626 (7.2)	2,611 (11.5)	4,003 (18.3)	7,056 (31.3)	<0.001
Hypertriglyceridemia (≥150 mg/dL)	16,348 (18.2)	0 (0.0)	0 (0.0)	32 (0.1)	16,316 (72.3)	<0.001
Low HDL cholesterol^b^ (*N* = 89,807)	20,039 (22.3)	792 (3.5)	2,150 (9.4)	4,973 (22.7)	12,124 (53.7)	<0.001
Hyper LDL cholesterol (≥159 mg/dL) (*N* = 89,806)	10,434 (11.6)	680 (3.0)	1,476 (6.5)	3,035 (13.9)	5,243 (23.2)	<0.001
Hyperglycemia after fasting (≥100 mg/dL)	14,297 (15.9)	640 (2.8)	1,965 (8.6)	3,735 (17.1)	7,957 (35.2)	<0.001
Metabolic syndrome	16,369 (18.2)	210 (0.9)	821 (3.6)	2,275 (10.4)	13,063 (57.9)	<0.001
HbA1c ≥ 6.5 (*N* = 89,805)	568 (0.6)	3 (0.0)	14 (0.1)	52 (0.2)	499 (2.2)	<0.001
MBP ≥83 (mmHg)	40,252 (44.8)	5,421 (24.0)	8,067 (35.4)	10,872 (49.7)	15,892 (70.4)	<0.001
PRISm	3,048 (3.4)	642 (2.8)	706 (3.1)	724 (3.3)	976 (4.3)	<0.001
SRP	2,917 (3.2)	654 (2.9)	687 (3.0)	679 (3.1)	897 (4.0)	<0.001
OP (FEV1(L)/FVC(L) ratio < LLN)	1,499 (1.7)	224 (1.0)	325 (1.4)	412 (1.9)	538 (2.4)	<0.001

Data are presented as the number of participants with percentages in parentheses.

We recorded the number of participants for whom clinical parameters were available. Unless otherwise indicated, data from 89,809 participants were available.

Detailed documentation of missing values for some variables was unavailable because our study utilized pre-existing health check-up data. However, the MCAR test result was not significant (*p* = 0.0827), indicating that the data were completely missing at random.

ALT, alanine aminotransferase; BMI, body mass index; BP, blood pressure; FEV1%, percent predicted forced expiratory volume in 1 s; FVC%, percent predicted forced vital capacity; HbA1c, hemoglobin A1c; HDL, high-density lipoprotein; LDL, low-density lipoprotein; LLN, lower limit of normal; MBP, mean blood pressure; MCAR, missing completely at random; OP, obstructive pattern; PRISm, preserved ratio impaired spirometry; SRP, spirometric restrictive pattern; TyG, triglyceride-glucose index.

MBP = diastolic BP + 0.33 × (average systolic BP − average diastolic BP).

*p* values from chi-square test or Fisher's exact test, as appropriate.

a High waist circumference was defined as ≥ 90 cm in males and ≥ 85 cm in females.

b Low HDL level was defined as <40 mg/dL in males and <50 mg/dL in females.

PRISm, SRP, and OP were observed in 3.4%, 3.2%, and 1.7% of participants, respectively. Among the total 3,048 PRISm participants, 1,521 (1.7%) were PRISm only and 1,527 (1.7%) presented with PRISm with SRP. The proportions of participants with impaired lung function also significantly increased across TyG quartiles (all *p* < 0.001). By contrast, regular exercise was more frequent in the lower TyG quartiles (*p* < 0.001).

### 3.3 Odds ratios (ORs) for lung dysfunction according to quartile of TyG

Multiple regression analyses were performed to evaluate the effects of TyG on lung dysfunction (Table 3 and Figure 2). In the fully adjusted logistic regression model (Model 6), higher TyG quartiles were associated with a stepwise increase in the odds of PRISm and SRP.

**Table 3:** Multiple logistic regression analysis of impaired lung function by quartile of TyG.

Types of impaired lung function	Q1 (≤7.97) [reference]	Q2 (7.98–8.33)	*p* value	Q3 (8.34–8.73)	*p* value	Q4 (≥8.74)	*p* value	*p* for trend
PRISm								
Model 1	1	1.340 (1.211–1.482)	<0.001	1.419 (1.276–1.578)	<0.001	1.530 (1.365–1.715)	<0.001	
								<0.001
Model 2	1	1.320 (1.193–1.461)	<0.001	1.383 (1.241–1.542)	<0.001	1.481 (1.317–1.666)	<0.001	
								<0.001
Model 3	1	1.161 (1.036–1.302)	0.01	1.206 (1.066–1.364)	0.003	1.292 (1.133–1.474)	<0.001	
								<0.001
Mode1 4	1	1.259 (1.133–1.399)	<0.001	1.305 (1.164–1.463)	<0.001	1.401 (1.239–1.585)	<0.001	
								<0.001
Model 5	1	1.320 (1.192–1.461)	<0.001	1.396 (1.255–1.554)	<0.001	1.506 (1.342–1.689)	<0.001	
								<0.001
Model 6	1	1.157 (1.030–1.299)	0.014	1.232 (1.085–1.398)	0.001	1.368 (1.193–1.567)	<0.001	
								<0.001
SRP								
Model 1	1	1.198 (1.095–1.311)	<0.001	1.248 (1.149–1.355)	<0.001	1.303 (1.195–1.421)	<0.001	
								<0.001
Model 2	1	1.187 (1.081–1.304)	<0.001	1.246 (1.46–1.355)	<0.001	1.289 (1.179–1.409)	<0.001	
								<0.001
Model 3	1	1.159 (1.054–1.274)	0.002	1.266 (1.138–1.408)	<0.001	1.295 (1.169–1.434)	<0.001	
								<0.001
Mode1 4	1	1.128 (1.023–1.244)	0.016	1.182 (1.083–1.289)	<0.001	1.216 (1.107–1.335)	<0.001	
								0.021
Model 5	1	1.242 (1.143–1.351)	<0.001	1.244 (1.139–1.358)	<0.001	1.321 (1.211–1.440)	<0.001	
								<0.001
Model 6	1	1.117 (1.014–1.230)	0.025	1.244 (1.120–1.382)	<0.001	1.246 (1.115–1.391)	<0.001	
								<0.001
OP								
Model 1	1	0.856 (0.724–1.019)	0.082	0.872 (0.732–1.038)	0.126	0.881 (0.742–1.048)	0.152	
								0.258
Model 2	1	0.854 (0.719–1.015)	0.073	0.871 (0.731–1.037)	0.12	0.873 (0.732–1.041)	0.13	
								0.214
Model 3	1	0.860 (0.724–1.021)	0.084	0.873 (0.733–1.039)	0.126	0.890 (0.735–1.077)	0.229	
								0.271
Mode1 4	1	0.848 (0.713–1.009)	0.063	0.865 (0.720–1.040)	0.123	0.869 (0.730–1.034)	0.114	
								0.179
Model 5	1	0.858 (0.723–1.019)	0.081	0.872 (0.733–1.038)	0.125	0.876 (0.737–1.041)	0.133	
								0.224
Model 6	1	0.841 (0.671–1.053)	0.131	0.859 (0.703–1.049)	0.136	0.888 (0.725–1.086)	0.246	
								0.135

Model 1: Adjusted for age, sex, smoking status, heavy alcohol consumption, regular exercise and MBP.

Model 2: Adjusted as in Model 1 plus WC.

Model 3: Adjusted as in Model 1 plus MS.

Model 4: Adjusted as in Model 1 plus lipid profile such as TC, LDL cholesterol and HDL cholesterol.

Model 5: Adjusted as in Model 1 plus HBA1c.

Model 6: Adjusted for age, sex, smoking status, heavy alcohol consumption, regular exercise, MS, and non-redundant, non-collinear covariates (VIF < 2.0; including HbA1c, ALT, total bilirubin, and creatinine).

ALT, alanine aminotransferase; HbA1c, hemoglobin A1c; LDL cholesterol, Low density lipoprotein cholesterol; LLN; MBP, mean blood pressure; OP, obstructive pattern; PRISm, preserved ratio impaired spirometry; SRP, spirmetric restrictive pattern; TC, Total cholesterol; TyG, triglyceride-glucose index; VIF, variance inflation factor.

MBP = diastolic BP + 0.33 × (average systolic BP − average diastolic BP).

LLN values were 81%, 82%, and 73% for FEV1%, FVC%, and FEV1(L)/FVC (L) ratio (%), respectively.

**Figure 2:**
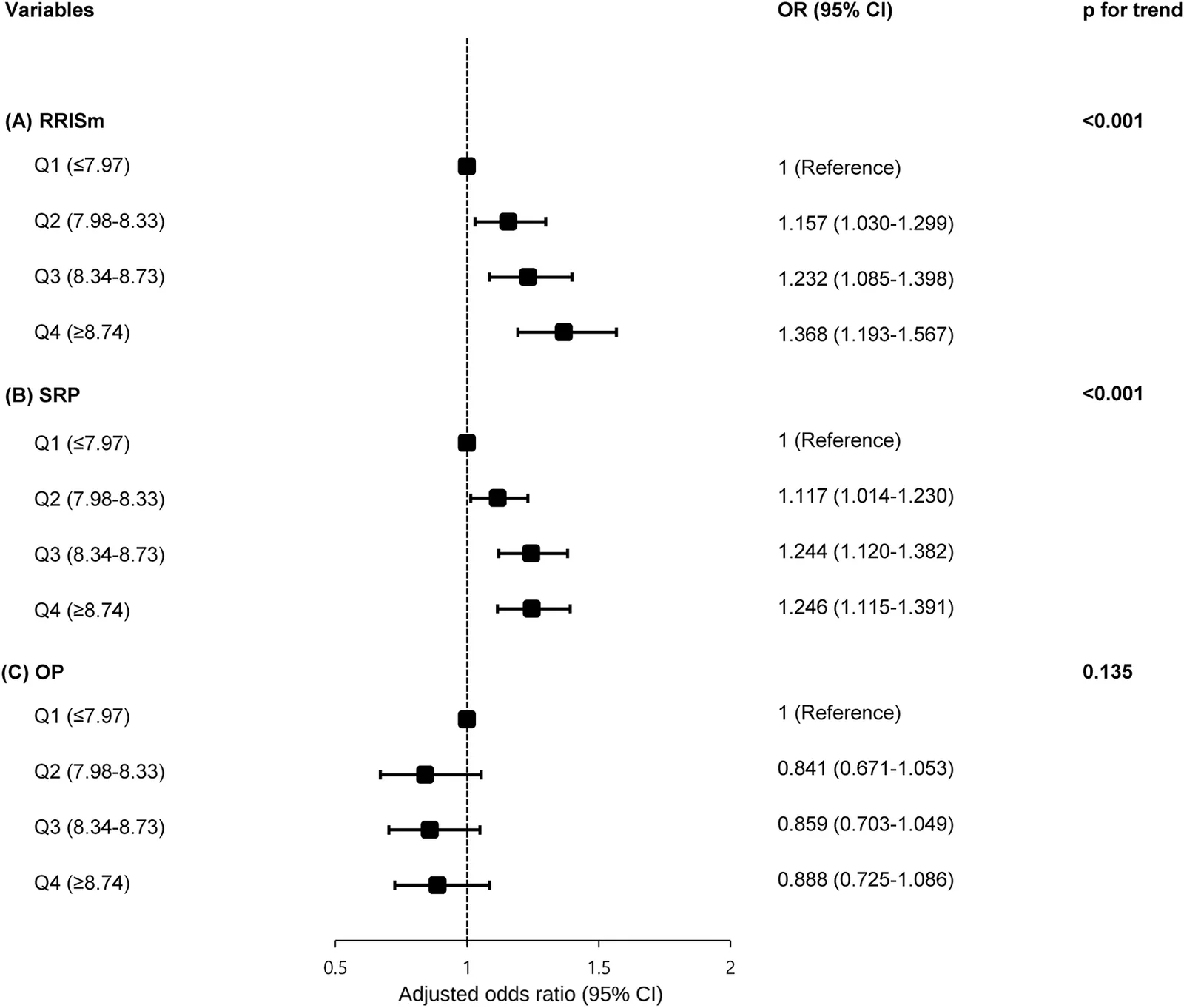
Multivariable-adjusted odds ratios for PRISm, SRP, and OP across TyG quartiles. Forest plots showing the multivariable-adjusted aORs with 95% CIs for **(A)** PRISm, **(B)** SRP, and **(C)** OP across TyG quartiles, using the lowest quartile (Q1) as the reference. Squares represent point estimates and horizontal lines represent 95% CIs; the dashed vertical line indicates the null effect (OR = 1.0). The *p* values for trend across quartiles are shown to the right of each panel. The aOR for PRISm **(A)** and SRP **(B)** increased with increasing TyG quartile (Q2-4). However, the differences in the aORs for OP among the groups were not consistently significant. The model was adjusted for age, sex, smoking status, heavy alcohol consumption, regular exercise, MS, and non-redundant, non-collinear covariates (VIF < 2.0; including HbA1c, ALT, total bilirubin, and creatinine). Individual components encompassed within the definition of MS (such as MBP, WC, and HDL-C) were explicitly excluded to eliminate multicollinearity. The LLN corresponded to 81%, 82%, and 73% for FEV_1_%, FVC%, and FEV_1_(L)/FVC ratio (%), respectively. ALT,Alanine Aminotransferase; aOR, adjusted odds ratio; CI, confidence interval; FEV1%, percent predicted forced expiratory volume in 1 s; FVC%, percent predicted forced vital capacity; HbA1c, Glycated hemoglobin; HDL-C, High-Density Lipoprotein Cholesterol; LLN, lower limit of normal; MBP, Mean Blood Pressure, MS, metabolic syndrome; OP, obstructive pattern; PRISm, preserved ratio impaired spirometry; SRP, spirometric restrictive pattern; TyG, triglyceride-glucose index; VIF, Variance Inflation Factor; WC, waist circumference.

Specifically, the aORs (95% CIs) for PRISm were 1.157(1.030–1.299), 1.232 (1.085–1.398), and 1.368 (1.193–1.567) in respective quartiles 2–4, TyG (*p* for trend < 0.001). Likewise, the aORs of SRP also increased across the TyG quartiles (*p* for trend < 0.001). However, no significant trend was observed for OP (*p* for trend = 0.135). When evaluating mutually exclusive outcome categories (Supplementary Table S3), higher TyG index quartiles (Q4 vs. Q1) remained significantly associated with increased risks of PRISm only [1.419 (1.198–1.681), *p* for trend <0.001], PRISm with SRP [1.525 (1.297–1.793), *p* for trend <0.001], and pure SRP [1.439 (1.222–1.693), *p* for trend = 0.009], whereas no significant association was observed for the OP [0.871 (0.732–1.037), *p* for trend = 0.165]. Sensitivity analyses employing conventional fixed-ratio criteria for both standard overlapping and mutually exclusive spirometric phenotypes yielded similar patterns of association with TyG (Supplementary Tables S1, S4). Furthermore, stratified subgroup analyses revealed that the positive association between TyG and PRISm risk was significantly more pronounced in males than in females, whereas no statistically significant interactions were observed across other clinical covariates (Supplementary Table S5).

## 4 Discussion

In the present study, higher TyG quartiles were associated with increased odds of PRISm and SRP, with a significant trend across quartiles, whereas no significant trend was observed for OP. These findings highlight a potential link between TyG and and lung function alterations such as PRISm and SRP, even in individuals without self-reported overt clinical disease. Additionally, these results extend previous Western observations (16, 19, 20, 27, 28) to an East Asian population, demonstrating a consistent association between the TyG and PRISm/SRP-type spirometric impairment across diverse populations.

The prevalence of PRISm in our cohort was 3.4%, which is substantially lower than that reported in previous studies. A recent meta-analysis reported a pooled prevalence of 12% (range: 4.5%–45.7%) with high heterogeneity (5). It varies markedly by race, geographical location, and study population (4, 5). However, our prevalence was also lower than that reported in Asian studies (5), possibly because of differences in population characteristics, although between-study heterogeneity complicates definitive interpretation (5). Most prior studies included individuals with preexisting CVMDs and chronic respiratory diseases (CRDs), which are associated with PRISm (3, 4, 7). Therefore, previous estimates may have overstated the prevalence of PRISm, whereas our sample of adults without overt CVMDs or CRDs likely reflects a lower PRISm burden.

To date, five studies have examined the association between IR and PRISm using various IR surrogate markers (16, 19, 20, 27, 28). While three studies specifically evaluated the TyG (16, 19, 20) -one of which additionally investigated estimated glucose disposal rate (eGDR) and other TyG-derived indices (20)- the remaining studies focused on alternative IR surrogates, including eGDR alone (28), and Homeostatic Model Assessment of Insulin Resistance (HOMA-IR) (27). Notably, all five studies were conducted in Western populations- (16, 27, 28) four utilized the US National Health and Nutrition Examination Survey (NHANES) dataset (16, 20, 27, 28) and one utilized the Austrian LEAD cohort (19). However, their findings have been inconsistent: two studies reported significant positive associations (16, 28), similar to our findings, whereas one found no association (27). Regarding TyG, two studies found no significant association between the TyG itself and PRISm risk (19, 20), although one of them demonstrated that TyG-derived indices were indeed significantly associated with PRISm (20). The remaining study reported an increased risk of PRISm associated with a higher TyG, this association reached only marginal statistical significance (*P* = 0.050) (19). Against this background, our study differs from prior studies in several key aspects with relevant clinical implications. First, this is the first study to be conducted in an East Asian population, to our knowledge. This is important, because metabolic characteristics, degree of IR, and prevalence of PRISm vary across ethnic groups (5, 17, 18). East Asians exhibit 30%–40% lower β-cell function than Caucasians, along with relatively higher insulin sensitivity (18). Moreover, Asians with comparable BMIs tended to have greater abdominal visceral fat (17). Second, we excluded participants with overt CVMDs and CRDs that could affect both IR (2, 9, 11, 14) and PRISm (3–7), unlike previous studies (19, 20, 27, 28). In addition, Zheng et al. did not address CVMDs or CRDs in their work (16). Collectively, underlying comorbidities-related systemic influences on PRISm and medication-induced alterations in TyG, can introduce substantial bias, potentially obscuring the true association between baseline IR and PRISm in previous reports. Third, we comprehensively evaluated lung function impairment using both internally derived lower limit of normal (LLN) criteria and conventional fixed cut-offs; the high consistency of findings across both definitions confirms that our conclusions are remarkably robust and free from definition-related bias. Fourth, we additionally performed sub-group analysis using mutually exclusive PRISm categories, given the moderate overlap and non-interchangeability between PRISm and SRP (6). Our analyses revealed that elevated TyG levels were associated with increased odds of PRISm only, SRP only, and the overlap phenotype (both PRISm and SRP). Although the adjusted odds ratio appeared slightly higher in the overlap group, these findings are derived from cross-sectional data and should be interpreted with caution. Further prospective studies are required to determine whether a synergistic interaction exists between PRISm and SRP in relation to metabolic burden. Finally, we and Zheng et al. (16) employed TyG as a surrogate marker of IR, in contrast to prior null findings based on Homeostatic Model Assessment of Insulin Resistance (HOMA-IR) (27). The TyG may better reflect muscle IR, whereas the HOMA-IR index mainly reflects hepatic IR (29). The link between muscle IR and impaired lung function may partly explain the discrepant findings across studies. Taken together, our findings carry meaningful epidemiological relevance by extending prior observations from Western populations to East Asians and supporting an independent positive association between TyG and PRISm.

Interestingly, our findings demonstrate that TyG exerts a statistically significant positive effect on the risk of both PRISm and SRP, with this association being notably more pronounced in males, unlike previous studies (20, 28). While a definitive mechanism underlying this observed sex difference remains to be fully elucidated, this observed disparity can primarily be attributed to distinct physiological profiles in body fat partitioning and sex hormone activity. Because the study cohort was relatively young (median age: 38.5 years), a majority of female participants were presumably premenopausal. Estrogen is well-recognized for bolstering insulin sensitivity and optimizing lipid dynamics, which may exert a buffering effect against metabolic stress-induced decline in lung function (30). Conversely, males are prone to greater visceral adiposity and inherently lower baseline insulin sensitivity, rendering them more vulnerable to the adverse respiratory consequences of TyG elevation (31). Thus, our findings underscore sex-specific mechanisms in metabolic-respiratory interactions within a younger demographic. However, future prospective research encompassing postmenopausal populations will be valuable to fully delineate the role of hormonal shifts in this relationship.

While previous studies primarily focused on PRISm, our evaluation extends to the spirometric restrictive pattern (SRP) and mutually exclusive spirometric phenotypes (Supplementary Tables S3, S4) Interestingly, individuals meeting criteria for PRISm with SRP exhibited the strongest association with TyG, suggesting a synergistic metabolic impact on combined reductions in FEV1 and FVC. However, interpreting SRP and pure SRP phenotypes in screening cohorts requires careful context. Traditionally, restrictive impairment is underrecognized in respiratory clinical practice. The latest European Respiratory Society/American Thoracic Society guidelines (24) define a normal FEV_1_/FVC and a reduced FVC and/or FEV_1_ as “nonspecific” pattern (if normal total lung capacity (TLC)), or restrictive impairment (if reduced TLC). However, plethysmographic TLC assessment was clinically and economically non-viable in our large-scale routine health-screening cohort. Consequently, the RP identified in our cohort is compatible with a spirometric restrictive pattern (SRP) rather than true restrictive lung disease. The SRP could be due to early obstructive or restrictive disease, small airway disease, or emphysema with air-trapping (32, 33) Furthermore, the potential inclusion of false-positive cases due to the absence of TLC measurements (such as submaximal inspiratory effort, or physiological variation) may have attenuated the strength of association between SRP and TyG compared with true restrictive lung disease. This likely accounts for the lower aOR observed for SRP than for PRISm in our primary analysis. Similarly, pure SRP consistently yielded lower aORs than PRISm with SRP in our mutually exclusive classification. Therefore, caution is required when interpreting the magnitude of association between TyG and SRP, particularly in cohorts or studies where TLC measurements are unavailable. Future studies incorporating body plethysmography are warranted to validate these phenotypical distinctions. From a pathophysiological standpoint, this structural resemblance between PRISm and SRP (3, 4) is highly consistent with the metabolic impact of TyG. Reflecting both lipotoxicity and glucotoxicity, an elevated TyG was associated with a parallel decline in FEV_1_ and FVC, thereby resulting in no net effect on the FEV_1_/FVC ratio (13). Therefore, the observed association between TyG and lung dysfunction, encompassing both PRISm and SRP, appears pathophysiologically plausible. Although the reason why IR is closely associated with PRISm and SRP, but not with OP, remains unclear, several hypothetical mechanisms may explain these findings. First, excess circulating free fatty acids (lipotoxicity) and hyperglycemia (glucotoxicity) in IR can promote oxidative stress, mitochondrial dysfunction, and chronic low-grade inflammation, contributing to the structural and functional impairment of the lung tissue (2, 8–10). Interestingly, IR and related conditions are more strongly associated with RP than OP (34–36). OP appears to be more closely linked to systemic inflammation (37) rather than IR (36). This suggests that IR primarily affects the lung parenchyma rather than the airway caliber, although its effects are closely associated with systemic inflammation (10). Second, the mechanical effects of obesity on IR may provide another possible explanation. IR is closely linked to obesity, particularly abdominal obesity (10), and is more strongly associated with SRP and PRISm than with OP (4, 38). Excess thoracoabdominal fat limits diaphragmatic movement and lung expansion, leading to decreased lung volume (39), typically with a greater decline in FVC than in FEV_1_, and a preserved or increased FEV_1_/FVC ratio. In contrast, the impact of obesity on OP may be underestimated, especially in healthy participants, because conventional spirometry is effort dependent and may mask the effects of obesity. Moreover, OP predominantly reflects the obstruction of large airways (40), which may further contribute to the underestimation of the functional debility of the airways using spirometry in healthy participants. However, PRISm appears to reflect small airway dysfunction (3, 4). This difference may further contribute to our findings; however, OP should be interpreted with caution when derived from screening spirometry, particularly in healthy young and middle-aged adults. Consequently, both metabolic and obesity-related mechanical effects on IR appear to contribute more to SRP and PRISm than to OP. Accordingly, TyG may serve as an integrative indicator of the metabolic processes underlying SRP and PRISm.

Our study has important clinical implications and strengths. Given the growing burden of PRISm (3–7) and the lack of established management strategies (4), our findings highlight the potential value of TyG in understanding the metabolic risk profile of individuals with PRISm. Notably, this is the first study in an Asian population, extending prior Western findings (16, 19, 28) (16, 28) and emphasizing the importance of considering metabolic health in the assessment of respiratory impairment. Additional strengths include a large sample sized population, and rigorous adjustment for multiple confounders. Furthermore, we conducted subgroup analyses using mutually exclusive outcome categories (PRISm only, PRISm with SRP, and pure SRP), considering the partial overlap and non-interchangeability between PRISm and spirometric restrictive impairment (6). This approach minimized potential misclassification bias and demonstrated consistent TyG associations across distinct phenotypes, thereby reinforcing the overall robustness of our findings. However, TyG thresholds vary across study populations and outcome definitions (14). Furthermore, metabolic profiles and the degree of IR differ substantially between racial and ethnic groups (17, 18). Therefore, further longitudinal research in this area is warranted.

Despite these strengths, our study has some limitations. First, its cross-sectional design precludes the determination of causal or directional relationships, emphasizing the importance of prospective research to evaluate directional associations and causality. Second, our study population was exclusively derived from a Korean health-screening cohort. Moreover, a substantial proportion of the initial cohort was excluded from the final analysis due to missing covariates or prespecified exclusion criteria (e.g., pre-existing chronic pulmonary diseases, diabetes, cardiovascular disease, or malignancies), resulting in systematic baseline differences between included and excluded participants (Supplementary Table S2). Although these strict eligibility criteria were methodologically essential to isolate the independent association between TyG and subclinical lung function impairment while minimizing major clinical confounders (2–12), it inevitably derived a relatively younger and metabolically healthier analytic population, introducing a potential selection bias or healthy-examinee effect and limitation of the external validity of our findings. Consequently, caution is warranted when generalizing these results to older individuals, populations with substantial comorbid conditions, or other ethnic groups. To overcome these limitations, prospective, multi-ethnic, and population-based longitudinal studies that include broader clinical spectrums are required to confirm our findings and evaluate their generalizability. Third, we cannot exclude the possibility of intra-individual changes in lung function and TyG levels. Transitions in PRISm status and TyG levels over time may have biased our results. Thus, further validation of how dynamic changes affect this relationship is required. Fourth, medical histories regarding underlying diseases were self-reported, which may have led to recall bias. Relatedly, although participants with known overt clinical diseases were excluded, our study cohort cannot be considered strictly “metabolically healthy” in a rigorous sense. Indeed, subclinical metabolic abnormalities were common, particularly among participants in the higher TyG quartiles. Unrecognized cardiovascular, metabolic, or respiratory diseases could still be present, and these subclinical conditions are known to be associated with both IR (2, 8–12) and PRISm (3–7). Therefore, our findings should be interpreted as representative of an apparently healthy population undergoing routine health screening rather than a strictly healthy reference group. In addition, residual or unrecognized confounding factors may have influenced our results despite adjustment for multiple potential confounders. Further prospective studies are required to confirm these findings. Finally, because previously established reference values for the Korean population were unavailable, our primary definition of LLN relied on the 5th percentile of the study population rather than a strictly pre-screened, healthy reference population. This approach might differ from the true 5th percentile derived from a strictly healthy Korean reference population (24) and could potentially influence absolute prevalence estimates of PRISm and SRP. Nevertheless, the consistency of our primary conclusions across sensitivity analyses utilizing both internally derived LLN criteria and fixed diagnostic cut-offs indicates that potential definition-related bias is minimal. However, further studies are needed to establish standardized reference equations based on large, healthy Korean cohorts to refine these prevalence estimates.

In conclusion, higher TyG index levels were independently associated with an increased prevalence of PRISm in individuals without self-reported overt clinical disease. These findings highlight the potential relevance of metabolic health in relation to spirometric impairment. However, longitudinal and prospective interventional studies are required to confirm these findings in diverse global populations.

## Data Availability

The datasets presented in this article are not readily available because due to ethical restrictions imposed by the Institutional Review Board of Kangbuk Samsung Hospital, the patient data are not available for distribution outside of the Kangbuk Samsung Hospital. (Data were not collected in a way that could be distributed widely). Requests to access the datasets should be directed to Surgeon Moon (suhyeon71moon@samsung.com) or the Institutional Review Board of Kangbuk Samsung Hospital (contact information below). - Address: 29 Saemunan-ro, Jongno-Gu, Seoul, Korea (03181) - irb. kbsmc@samsung.com - Telephone: 82-2-2001-1943, 1945 - Fax: 82-2-2001-1946.
