# Supplementary material for: Triglyceride–glucose index and preserved ratio impaired spirometry in 89,809 individuals without self-reported overt clinical disease
Source: Front Med (Lausanne). 2026 Sep 25;13:1880988. doi: 10.3389/fmed.2026.1880988 (PMC13649719; doi:10.3389/fmed.2026.1880988)
Supplement: Supplementary file 1 [file Table1.docx]

Supplementary Material

**Supplementary Table S1.** Sensitivity analysis of multiple logistic regression for impaired lung function according to TyG quartiles using conventional fixed spirometric criteria.

**Supplementary Table 2.** Comparison of baseline characteristics between participants included in and excluded from the final analysis.

**Supplementary Table 3.** Distribution and Clinical Characteristics of Participants According to Mutually Exclusive Spirometric Phenotypes Based on Lower Limit of Normal (LLN) Criteria.

**Supplementary Table 4.** Distribution and Clinical Characteristics of Participants According to Mutually Exclusive Spirometric Phenotypes Based on Fixed Ratio Criteria.

**Supplementary Table 5.** Subgroup Analyses of the Association Between TyG Index and PRISm (LLN criteria) Stratified by Key Clinical Characteristics.

# Supplementary Table 1. Sensitivity analysis of multiple logistic regression for impaired lung function according to TyG quartiles using conventional fixed spirometric criteria.

|  | **Q1 (≤7.97) [reference]** | **Q2 (7.98-8.33)** | ***p* value** | **Q3 (8.34-8.73)** | ***p* value** | **Q4 (≥8.74)** | ***p* value** | ***p* for trend** |
| --- | --- | --- | --- | --- | --- | --- | --- | --- |
| **PRISm** |  |  |  |  |  |  |  |  |
| Model 1 | 1 | 1.330 (1.206-1.466) | <0.001 | 1.399 (1.263-1.550) | <0.001 | 1.474 (1.321-1.643) | <0.001 |  |
|  |  |  |  |  |  |  |  | <0.001 |
| Model 2 | 1 | 1.391 (1.232-1.570) | <0.001 | 1.463 (1.285-1.666) | <0.001 | 1.511 (1.313-1.737) | <0.001 |  |
|  |  |  |  |  |  |  |  | <0.001 |
| Model 3 | 1 | 1.202 (1.048-1.378) | 0.008 | 1.246 (1.075-1.444) | 0.003 | 1.284 (1.097-1.502) | 0.002 |  |
|  |  |  |  |  |  |  |  | 0.004 |
| Mode1 4 | 1 | 1.320 (1.166-1.495) | <0.001 | 1.372 (1.200-1.569) | <0.001 | 1.417 (1.228-1.636) | <0.001 |  |
|  |  |  |  |  |  |  |  | <0.001 |
| Model 5 | 1 | 1.377 (1.220-1.554) | <0.001 | 1.457 (1.282-1.656) | <0.001 | 1.512 (1.319-1.734) | <0.001 |  |
|  |  |  |  |  |  |  |  | <0.001 |
| Model 6 | 1 | 1.130 (1.028-1.243) | 0.011 | 1.230 (1.109-1.364) | <0.001 | 1.283 (1.149-1.433) | <0.001 |  |
|  |  |  |  |  |  |  |  | <0.001 |
| **SRP** |  |  |  |  |  |  |  |  |
| Model 1 | 1 | 1.166 (1.063-1.278) | <0.001 | 1.236 (1.137-1.344) | <0.001 | 1.272 (1.165-1.389) | <0.001 |  |
|  |  |  |  |  |  |  |  | <0.001 |
| Model 2 | 1 | 1.264 (1.162-1.375) | <0.001 | 1.394 (1.273-1.528) | <0.001 | 1.459 (1.321-1.610) | <0.001 |  |
|  |  |  |  |  |  |  |  | <0.001 |
| Model 3 | 1 | 1.144 (1.041-1.258) | 0.005 | 1.250 (1.128-1.385) | <0.001 | 1.305 (1.169-1.456) | <0.001 |  |
|  |  |  |  |  |  |  |  | <0.001 |
| Mode1 4 | 1 | 1.207 (1.107-1.315) | <0.001 | 1.314 (1.196-1.444) | <0.001 | 1.372 (1.240-1.518) | <0.001 |  |
|  |  |  |  |  |  |  |  | <0.001 |
| Model 5 | 1 | 1.258 (1.156-1.367) | <0.001 | 1.392 (1.272-1.523) | <0.001 | 1.460 (1.326-1.608) | <0.001 |  |
|  |  |  |  |  |  |  |  | <0.001 |
| Model 6 | 1 | 1.176 (1.025-1.349) | 0.021 | 1.212 (1.045-1.406) | 0.011 | 1.248 (1.065-1.461) | 0.006 |  |
|  |  |  |  |  |  |  |  | 0.011 |
| **OP** |  |  |  |  |  |  |  |  |
| Model 1 | 1 | 0.864 (0.728-1.025) | 0.094 | 0.874 (0.734-1.040) | 0.128 | 0.888 (0.747-1.055) | 0.178 |  |
|  |  |  |  |  |  |  |  | 0.305 |
| Model 2 | 1 | 0.858 (0.722-1.020) | 0.082 | 0.871 (0.732-1.037) | 0.121 | 0.876 (0.735-1.045) | 0.141 |  |
|  |  |  |  |  |  |  |  | 0.236 |
| Model 3 | 1 | 0.864 (0.727-1.026) | 0.095 | 0.873 (0.734-1.039) | 0.127 | 0.894 (0.785-1.081) | 0.247 |  |
|  |  |  |  |  |  |  |  | 0.299 |
| Mode1 4 | 1 | 0.869 (0.731-1.033) | 0.111 | 0.875 (0.735-1.041) | 0.132 | 0.903 (0.753-1.082) | 0.27 |  |
|  |  |  |  |  |  |  |  | 0.41 |
| Model 5 | 1 | 0.862 (0.726-1.024) | 0.091 | 0.873 (0.733-1.039) | 0.126 | 0.880 (0.740-1.045) | 0.145 |  |
|  |  |  |  |  |  |  |  | 0.249 |
| Model 6 | 1 | 0.856 (0.721-1.017) | 0.077 | 0.870 (0.719-1.053) | 0.152 | 0.872 (0.733-1.038) | 0.124 |  |
|  |  |  |  |  |  |  |  | 0.181 |

PRISm was defined as FEV1/FVC ≥ 0.7 and FEV1<80% predicted. SRP was defined as FEV1/FVC ≥ 0.7 and FVC <80% predicted, and OP was defined as FEV1/FVC <0.7.

Model 1: Adjusted for age, sex, smoking status, heavy alcohol consumption, regular exercise and MBP.

Model 2: Adjusted as in Model 1 plus WC.

Model 3: Adjusted as in Model 1 plus MS.

Model 4: Adjusted as in Model 1 plus lipid profile such as TC, LDL cholesterol and HDL cholesterol.

Model 5: Adjusted as in Model 1 plus HBA1c.

Model 6: Adjusted for age, sex, smoking status, heavy alcohol consumption, regular exercise, MS, and non-redundant, non-collinear covariates (VIF < 2.0; including HbA1c, ALT, total bilirubin, and creatinine).

**Abbreviations:** ALT, alanine aminotransferase; HbA1c, hemoglobin A1c; LDL cholesterol, Low density lipoprotein cholesterol; LLN; MBP, mean blood pressure; OP, obstructive pattern; PRISm, preserved ratio impaired spirometry; SRP, spirometric restrictive pattern; TC, Total cholesterol; TyG, triglyceride-glucose index; VIF, variance inflation factor.

MBP*=* diastolic BP + 0.33 × (average systolic BP - average diastolic BP).

# Supplementary Table 2. Comparison of baseline characteristics between participants included in and excluded from the final analysis.

| **Characteristics** | **Excluded participants**  **(n = 124,742)** | **Included participants**  **(n = 89,809)** | **Absolute SMD** | ***p* value** |
| --- | --- | --- | --- | --- |
| Age (years) | 44.25 ± 9.12 | 38.50 ± 7.35 | 0.694 | <0.001 |
| Sex (male), n (%) | 72,983 (58.5) | 46,739 (52.0) | 0.13 | <0.001 |
| Waist circumference (cm) | 83.21 ± 10.01 | 80.23 ± 9.48 | 0.306 | <0.001 |
| BMI (kg/m²) | 24.13 ± 3.55 | 23.23 ± 3.26 | 0.264 | <0.001 |
| Smoking (pack-years) | 5.36 ± 9.22 (n = 120,453) | 3.13 ± 6.50 (n = 89,706) | 0.28 | <0.001 |
| Alcohol consumption (g/day) | 14.80 ± 23.15 (n = 109,047) | 12.98 ± 20.46 | 0.083 | <0.001 |
| Moderate physical activity frequency (times/week) | 0.87 ± 1.46 | 0.85 ± 1.41 | 0.014 | 0.007 |
| Total cholesterol (mg/dL) | 197.04 ± 36.64 | 192.46 ± 31.11 | 0.135 | <0.001 |
| Triglyceride (mg/dL) | 105.0 (73–157) | 89.0 (64–130) | 0.25 | <0.001 |
| HDL cholesterol (mg/dL) | 58.37 ± 16.30 | 62.17 ± 16.63 | 0.231 | <0.001 |
| LDL cholesterol (mg/dL) | 129.14 ± 34.64 | 123.49 ± 30.30 | 0.174 | <0.001 |
| Fasting glucose (mg/dL) | 97.83 ± 17.77 | 93.36 ± 10.65 | 0.305 | <0.001 |
| Systolic BP (mmHg) | 111.31 ± 13.17 | 108.20 ± 11.94 | 0.247 | <0.001 |
| Diastolic BP (mmHg) | 72.57 ± 10.17 | 69.87 ± 9.38 | 0.276 | <0.001 |
| Metabolic syndrome, n (%) | 49,602 (39.8) | 16,369 (18.2) | 0.489 | <0.001 |
| TyG index | 8.57 ± 0.62 (n=124,737) | 8.34 ± 0.57 | 0.334 | <0.001 |
| Measured FEV1 (L) | 3.17 ± 0.69 (n=123,673) | 3.30 ± 0.68 | 0.19 | <0.001 |
| FEV1% | 95.33 ± 11.56 (n=123,638) | 98.00 ± 10.71 | 0.238 | <0.001 |
| Measured FVC (L) | 3.91 ± 0.87 (n=123,673) | 4.00 ± 0.87 | 0.103 | <0.001 |
| FVC% | 95.31 ± 11.35 (n=123,638) | 98.31 ± 10.69 | 0.271 | <0.001 |
| FEV1(L)/FVC(L) ratio | 0.813 ± 0.061 (n=123,671) | 0.829 ± 0.059 | 0.266 | <0.001 |

Data are presented as mean ± standard deviation, median (interquartile range), or number (percentage), as appropriate. Statistics were calculated using available observations for each variable; therefore, denominators may vary because of missing data. Available sample sizes are shown for smoking and alcohol consumption because of the relatively greater proportion of missing observations. P-values were calculated using Welch’s t-test for continuous variables, except for triglyceride levels, which were compared using the Mann–Whitney U test, and using the Pearson chi-square test for categorical variables. Absolute standardized mean differences (SMDs) are presented to quantify the magnitude of between-group differences. The SMD for triglyceride levels was calculated using the untransformed means and standard deviations.

**Abbreviations:** BMI, body mass index; HDL, high-density lipoprotein; LDL, low-density lipoprotein; BP, blood pressure; TyG, triglyceride-glucose index; FEV1, forced expiratory volume in 1 s; FVC, forced vital capacity; SMD, standardized mean difference.

**Supplementary Table 3. Distribution and Clinical Characteristics of Participants According to Mutually Exclusive Spirometric Phenotypes Based on Lower Limit of Normal (LLN) Criteria.**

|  | **Q1 (≤7.97) [reference]** | ***p* value** | **Q2 (7.98-8.33)** | ***p* value** | **Q3 (8.34-8.73)** | ***p* value** | **Q4 (≥8.74)** | ***p* value** | ***p* for trend** |
| --- | --- | --- | --- | --- | --- | --- | --- | --- | --- |
| **PRISm only** |  |  |  |  |  |  |  |  |  |
| Model 1 | 1 |  | 1.174 (1.018-1.354) | 0.027 | 1.263 (1.087-1.467) | 0.002 | 1.470 (1.249-1.731) | <0.001 |  |
|  |  |  |  |  |  |  |  |  | <0.001 |
| Model 2 | 1 |  | 1.162 (1.006-1.341) | 0.041 | 1.240 (1.064-1.444) | 0.006 | 1.436 (1.2145-1.698) | <0.001 |  |
|  |  |  |  |  |  |  |  |  | <0.001 |
| Model 3 | 1 |  | 1.208 (1.029-1.419) | 0.021 | 1.266 (1.063-1.507) | 0.008 | 1.326 (1.102-1.596) | 0.003 |  |
|  |  |  |  |  |  |  |  |  | 0.005 |
| Model 4 | 1 |  | 1.176 (1.019-1.357) | 0.026 | 1.266 (1.086-1.475) | 0.003 | 1.451 (1.228-1.715) | <0.001 |  |
|  |  |  |  |  |  |  |  |  | <0.001 |
| Model 5 | 1 |  | 1.165 (1.010-1.344) | 0.036 | 1.250 (1.075-1.454) | 0.004 | 1.433 (1.217-1.687) | <0.001 |  |
|  |  |  |  |  |  |  |  |  | <0.001 |
| Model 6 | 1 |  | 1.165 (1.0008-1.346) | 0.039 | 1.236 (1.059-1.443) | 0.007 | 1.419 (1.198-1.681) | <0.001 |  |
|  |  |  |  |  |  |  |  |  | <0.001 |
| **PRISm with SRP** |  |  |  |  |  |  |  |  |  |
| Model 1 | 1 |  | 1.506 (1.309-1.732) | <0.001 | 1.596 (1.377-1.849) | <0.001 | 1.634 (1.398-1.910) | <0.001 |  |
|  |  |  |  |  |  |  |  |  | <0.001 |
| Model 2 | 1 |  | 1.470 (1.275-1.693) | <0.001 | 1.531 (1.317-1.781) | <0.001 | 1.550 (1.318-1.822) | <0.001 |  |
|  |  |  |  |  |  |  |  |  | <0.001 |
| Model 3 | 1 |  | 1.1183 (1.025-1.365) | 0.021 | 1.280 (1.110-1.488) | 0.001 | 1.484 (1.260-1.747) | <0.001 |  |
|  |  |  |  |  |  |  |  |  | <0.001 |
| Model 4 | 1 |  | 1.367 (1.180- 1.584) | <0.001 | 1.398 (1.191-1.640) | <0.001 | 1.416 (1.193-1.681) | <0.001 |  |
|  |  |  |  |  |  |  |  |  | <0.001 |
| Model 5 | 1 |  | 1.469 (1.274-1.693) | <0.001 | 1.553 (1.336-1.804) | <0.001 | 1.589 (1.353-1.864) | <0.001 |  |
|  |  |  |  |  |  |  |  |  | <0.001 |
| Model 6 | 1 |  | 1.432 (1.242-1.652) | <0.001 | 1.500 (1.288-1.745) | <0.001 | 1.525 (1.297-1.793) | <0.001 |  |
|  |  |  |  |  |  |  |  |  | <0.001 |
| **Pure SRP** |  |  |  |  |  |  |  |  |  |
| Model 1 | 1 |  | 1.223 (1.042-1.436) | 0.014 | 1.256 (1.081-1.459) | 0.003 | 1.345 (1.150-1.574) | <0.001 |  |
|  |  |  |  |  |  |  |  |  | <0.010 |
| Model 2 | 1 |  | 1.207 (1.013-1.437) | 0.035 | 1.235 (1.061-1.437) | 0.006 | 1.334 (1.135-1.569) | <0.001 |  |
|  |  |  |  |  |  |  |  |  | 0.018 |
| Model 3 | 1 |  | 1.261 (1.086-1.465) | 0.002 | 1.370 (1.169-1.605) | <0.001 | 1.374 (1.134-1.664) | 0.001 |  |
|  |  |  |  |  |  |  |  |  | <0.001 |
| Model 4 | 1 |  | 1.237 (1.047-1.461) | 0.013 | 1.328 (1.134-1.555) | <0.001 | 1.414 (1.200-1.667) | <0.001 |  |
|  |  |  |  |  |  |  |  |  | 0.025 |
| Model 5 | 1 |  | 1.260 (1.068-1.486) | 0.006 | 1.331 (1.137-1.559) | <0.001 | 1.434 (1.218-1.688) | <0.001 |  |
|  |  |  |  |  |  |  |  |  | 0.012 |
| Model 6 | 1 |  | 1.267 (1.075-1.493) | 0.005 | 1.335 (1.141-1.563) | <0.001 | 1.439 (1.222-1.693) | <0.001 |  |
|  |  |  |  |  |  |  |  |  | 0.009 |
| **OP** |  |  |  |  |  |  |  |  |  |
| Model 1 | 1 |  | 0.842 (0.710-0.999) | 0.049 | 0.850 (0.717-1.008) | 0.062 | 0.864 (0.726-1.029) | 0.101 |  |
|  |  |  |  |  |  |  |  |  | 0.108 |
| Model 2 | 1 |  | 0.854 (0.7169-1.015) | 0.073 | 0.871 (0.731-1.037) | 0.12 | 0.873 (0.732-1.041) | 0.13 |  |
|  |  |  |  |  |  |  |  |  | 0.214 |
| Model 3 | 1 |  | 0.876 (0.738-1.041) | 0.132 | 0.882 (0.741-1.050) | 0.159 | 0.908 (0.750-1.099) | 0.323 |  |
|  |  |  |  |  |  |  |  |  | 0.386 |
| Model 4 | 1 |  | 0.848 (0.713-1.009) | 0.063 | 0.865 (0.720-1.040) | 0.123 | 0.869 (0.730-1.034) | 0.114 |  |
|  |  |  |  |  |  |  |  |  | 0.179 |
| Model 5 | 1 |  | 0.858 (0.723-1.019) | 0.081 | 0.872 (0.733-1.038) | 0.125 | 0.876 (0.737-1.041) | 0.133 |  |
|  |  |  |  |  |  |  |  |  | 0.224 |
| Model 6 | 1 |  | 0.852 (0.717-1.011) | 0.067 | 0.867 (0.716-1.049) | 0.143 | 0.871 (0.732-1.037) | 0.122 |  |
|  |  |  |  |  |  |  |  |  | 0.165 |

Model 1: Adjusted for age, sex, smoking status, heavy alcohol consumption, regular exercise and MBP.

Model 2: Adjusted as in Model 1 plus WC.

Model 3: Adjusted as in Model 1 plus MS.

Model 4: Adjusted as in Model 1 plus lipid profile such as TC, LDL cholesterol and HDL cholesterol.

Model 5: Adjusted as in Model 1 plus HBA1c.

Model 6: Adjusted for age, sex, smoking status, heavy alcohol consumption, regular exercise, MS, and non-redundant, non-collinear covariates (VIF < 2.0; including HbA1c, ALT, total bilirubin, and creatinine).

ALT, alanine aminotransferase; HbA1c, hemoglobin A1c; LDL cholesterol, Low density lipoprotein cholesterol; LLN; MBP, mean blood pressure; OP, obstructive pattern; PRISm, preserved ratio impaired spirometry; SRP, spirmetric restrictive pattern; TC, Total cholesterol; TyG, triglyceride-glucose index; VIF, variance inflation factor.

MBP = diastolic BP + 0.33 × (average systolic BP - average diastolic BP).

LLN values were 81%, 82%, and 73% for FEV1%, FVC%, and FEV1(L)/FVC (L) ratio (%), respectively.

**Supplementary Table 4. Distribution and Clinical Characteristics of Participants According to Mutually Exclusive Spirometric Phenotypes Based on Fixed Ratio Criteria.**

|  | **Q1 (≤7.97) [reference]** | ***p* value** | **Q2 (7.98-8.33)** | ***p* value** | **Q3 (8.34-8.73)** | ***p* value** | **Q4 (≥8.74)** | ***p* value** | ***p* for trend** |
| --- | --- | --- | --- | --- | --- | --- | --- | --- | --- |
| **PRISm only** |  |  |  |  |  |  |  |  |  |
| Model 1 | 1 |  | 1.193 (1.013-1.403) | 0.034 | 1.245 (1.051-1.473) | 0.011 | 1.347 (1.126-1.610) | 0.001 |  |
|  |  |  |  |  |  |  |  |  | 0.001 |
| Model 2 | 1 |  | 1.221 (1.024-1.455) | 0.026 | 1.230 (1.029-1.471) | 0.023 | 1.414 (1.169-1.710) | <0.001 |  |
|  |  |  |  |  |  |  |  |  | <0.001 |
| Model 3 | 1 |  | 1.181 (1.030-1.354) | 0.017 | 1.221 (1.054-1.415) | 0.008 | 1.262 (1.079-1.476) | 0.004 |  |
|  |  |  |  |  |  |  |  |  | 0.007 |
| Model 4 | 1 |  | 1.192 (1.012-1.405) | 0.036 | 1.247 (1.049-1.483) | 0.012 | 1.353 (1.124-1.628) | 0.001 |  |
|  |  |  |  |  |  |  |  |  | 0.002 |
| Model 5 | 1 |  | 1.206 (1.023-1.421) | 0.026 | 1.282 (1.078-1.524) | 0.005 | 1.423 (1.182-1.714) | <0.001 |  |
|  |  |  |  |  |  |  |  |  | <0.001 |
| Model 6 | 1 |  | 1.203 (1.021-1.417) | 0.027 | 1.268 (1.068-1.506) | 0.007 | 1.407 (1.170-1.692) | <0.001 |  |
|  |  |  |  |  |  |  |  |  | <0.001 |
| **PRISm with SRP** |  |  |  |  |  |  |  |  |  |
| Model 1 | 1 |  | 1.397 (1.239-1.576) | <0.001 | 1.481 (1.304-1.682) | <0.001 | 1.539 (1.343-1.764) | <0.001 |  |
|  |  |  |  |  |  |  |  |  | <0.001 |
| Model 2 | 1 |  | 1.383 (1.225-1.561) | <0.001 | 1.456 (1.279-1.658) | <0.001 | 1.506 (1.309-1.732) | <0.001 |  |
|  |  |  |  |  |  |  |  |  | <0.001 |
| Model 3 | 1 |  | 1.191 (1.012-1.403) | 0.036 | 1.245 (1.049-1.478) | 0.012 | 1.351 (1.125-1.622) | 0.001 |  |
|  |  |  |  |  |  |  |  |  | 0.002 |
| Model 4 | 1 |  | 1.423 (1.262-1.604) | <0.001 | 1.535 (1.352-1.742) | <0.001 | 1.592 (1.391-1.822) | <0.001 |  |
|  |  |  |  |  |  |  |  |  | <0.001 |
| Model 5 | 1 |  | 1.380 (1.223-1.556) | <0.001 | 1.478 (1.301-1.679) | <0.001 | 1.527 (1.333-1.749) | <0.001 |  |
|  |  |  |  |  |  |  |  |  | <0.001 |
| Model 6 | 1 |  | 1.301 (1.130-1.394) | <0.001 | 1.405 (1.198-1.642) | <0.001 | 1.472 (1.228-1.753) | <0.001 |  |
|  |  |  |  |  |  |  |  |  | <0.001 |
| **Pure SRP** |  |  |  |  |  |  |  |  |  |
| Model 1 | 1 |  | 1.138 (1.005-1.289) | 0.042 | 1.179 (1.048-1.327) | 0.006 | 1.224 (1.083-1.382) | 0.001 |  |
|  |  |  |  |  |  |  |  |  | 0.042 |
| Model 2 | 1 |  | 1.146 (1.002-1.311) | 0.047 | 1.171 (1.040-1.318) | 0.009 | 1.214 (1.072-1.374) | 0.002 |  |
|  |  |  |  |  |  |  |  |  | 0.037 |
| Model 3 | 1 |  | 1.173 (1.031-1.335) | 0.016 | 1.239 (1.100-1.35) | <0.001 | 1.301 (1.148-1.473) | <0.001 |  |
|  |  |  |  |  |  |  |  |  | 0.017 |
| Model 4 | 1 |  | 1.205 (1.022-1.420) | 0.027 | 1.266 (1.064-1.505) | 0.008 | 1.377 (1.144-1.658) | <0.001 |  |
|  |  |  |  |  |  |  |  |  | <0.001 |
| Model 5 | 1 |  | 1.186 (1.039-1.353) | 0.012 | 1.209 (1.072-1.364) | 0.002 | 1.283 (1.130-1.456) | <0.001 |  |
|  |  |  |  |  |  |  |  |  | 0.012 |
| Model 6 | 1 |  | 1.246 (1.106-1.404) | <0.001 | 1.259 (1.105-1.434) | <0.001 | 1.343 (1.185-1.522) | <0.001 |  |
|  |  |  |  |  |  |  |  |  | <0.001 |
| **OP** |  |  |  |  |  |  |  |  |  |
| Model 1 | 1 |  | 0.901 (0.670-1.212) | 0.491 | 0.971 (0.720-1.309) | 0.846 | 1.127 (0.825-1.539) | 0.453 |  |
|  |  |  |  |  |  |  |  |  | 0.47 |
| Model 2 | 1 |  | 0.877 (0.652-1.179) | 0.384 | 0.961 (0.713-1.294) | 0.793 | 1.101 (0.803-1.509) | 0.55 |  |
|  |  |  |  |  |  |  |  |  | 0.591 |
| Model 3 | 1 |  | 0.872 (0.789-0.963) | 0.007 | 0.926 (0.825-1.039) | 0.19 | 0.943 (0.853-1.042) | 0.246 |  |
|  |  |  |  |  |  |  |  |  | 0.093 |
| Model 4 | 1 |  | 0.872 (0.788-0.964) | 0.008 | 0.929 (0.823-1.048) | 0.231 | 0.942 (0.852-1.041) | 0.24 |  |
|  |  |  |  |  |  |  |  |  | 0.102 |
| Model 5 | 1 |  | 0.894 (0.667-1.200) | 0.456 | 0.965 (0.717-1.299) | 0.815 | 1.140 (0.840-1.547) | 0.400 |  |
|  |  |  |  |  |  |  |  |  | 0.398 |
| Model 6 | 1 |  | 0.819 (0.610-1.101) | 0.186 | 0.939 (0.697-1.265) | 0.678 | 1.008 (0.734-1.383) | 0.962 |  |
|  |  |  |  |  |  |  |  |  | 0.928 |

PRISm was defined as FEV1/FVC ≥ 0.7 and FEV1<80% predicted. SRP was defined as FEV1/FVC ≥ 0.7 and FVC <80% predicted, and OP was defined as FEV1/FVC <0.7.

Model 1: Adjusted for age, sex, smoking status, heavy alcohol consumption, regular exercise and MBP.

Model 2: Adjusted as in Model 1 plus WC.

Model 3: Adjusted as in Model 1 plus MS.

Model 4: Adjusted as in Model 1 plus lipid profile such as TC, LDL cholesterol and HDL cholesterol.

Model 5: Adjusted as in Model 1 plus HBA1c.

Model 6: Adjusted for age, sex, smoking status, heavy alcohol consumption, regular exercise, MS, and non-redundant, non-collinear covariates (VIF < 2.0; including HbA1c, ALT, total bilirubin, and creatinine).

**Abbreviations:** ALT, alanine aminotransferase; HbA1c, hemoglobin A1c; LDL cholesterol, Low density lipoprotein cholesterol; LLN; MBP, mean blood pressure; OP, obstructive pattern; PRISm, preserved ratio impaired spirometry; SRP, spirometric restrictive pattern; TC, Total cholesterol; TyG, triglyceride-glucose index; VIF, variance inflation factor.

MBP*=* diastolic BP + 0.33 × (average systolic BP - average diastolic BP).

**Supplementary Table 5. Subgroup Analyses of the Association Between TyG Index and PRISm (LLN criteria) Stratified by Key Clinical Characteristics.**

|  | **No** | **PRISm : aOR** | ***p* value** | **SRP: aOR** | ***p* value** | **OP : aOR** | ***p* value** |
| --- | --- | --- | --- | --- | --- | --- | --- |
| **sex** | Male (n=46,739, 52.0%) | 1.421 (1.100-1.836) *p=*0.007 | <0.001 | 1.490 (1.077-1.718) *p=*0.026 | 0.007 | 1.045 (0.978-1.116) *p=*0.197 | 0.195 |
|  | Female (n=43,070, 48.0%) | 1.109 (0.990-1.242) *p=*0.073 |  | 1.233 (1.129-1.325) *p<*0.001 |  | 0.945 (0.874-1.021) *p=*0.153 |  |
| **Age** | Age<39 (n=50,448, 56.2%) | 1.401 (1.210-1.615) *p<*0.001 | 0.156 | 1.224 (1.009-1.486) *p=*0.038 | 0.735 | 0.960 (0.884-1.043) *p=*0.338 | 0.004 |
|  | Age≥39 (n=39,361, 43.8%) | 1.416 (1.238-1.614) *p*<0.001 |  | 1.271 (1.011-1.599) *p=*0.040 |  | 0.983 (0.866-1.117) *p=*0.798 |  |
| **Smoking** | Non-smoker (n=55,460, 61.8%) | 1.530 (1.337-1.752) *p<*0.001 | 0.052 | 1.437 (1.150-1.795) *p=*0.001 | 0.108 | 0.965 (0.853-1.092) *p=*0.575 | 0.951 |
|  | Forer smoker (n= 20,574, 22.9%) | 1.552 (1.247-1.932) *p<*0.001 |  | 1.429 (1.068-1.913) *p=*0.016 |  | 0.969 (0.654-1.437) *p=*0.876 |  |
|  | Current smoker (n=13,775, 15.3%) | 1.892 (1.456-2.460) *p<*0.001 |  | 1.513 (1.108-2.066) *p=*0.009 |  | 1.014 (0.785-1.309) *p=*0.918 |  |
| **Obesity** | BMI<25 (n=65,828, 73.3%) | 1.463 (1.211-1.767) *p<*0.001 | 0.609 | 1.149 (1.002-1.316) *p=*0.046 | 0.198 | 0.977 (0.805-1.164) *p=*0.811 | 0.637 |
|  | BMI≥25 (n= 23,981, 26.7%) | 1.601 (1.283-1.997) *p<*0.001 |  | 1.199 (1.023-1.406) *p=*0.025 |  | 0.923 (0.814-1.047) *p=*0.212 |  |
| **MS** | No (n = 73,340, 81.8%) | 1.182 (1.054-1.293) *p=*0.007 | 0.056 | 1.175 (1.032-1.339) *p=*0.015 | 0.125 | 0.953 (0.798-1.139) *p=*0.597 | 0.488 |
|  | Yes (n = 16,369, 18.2%) | 1.224 (1.097-1.333) *p=*0.001 |  | 1.252 (1.100-1.425) *p*<0.001 |  | 1.119 (0.968-1.294) *p=*0.128 |  |

Adjusted odds ratio and 95% CIs were analyzed using binary logistic regression. Model was adjusted for age, sex, smoking status, heavy alcohol consumption, regular exercise, MS, and non-redundant, non-collinear covariates (VIF < 2.0; including HbA1c, ALT, total bilirubin, and creatinine). The variable used to divide subgroup was excluded from this analysis.

ALT, alanine aminotransferase; HbA1c, hemoglobin A1c; MS, metabolic syndrome; OP, obstructive pattern; PRISm, preserved ratio impaired spirometry; SRP, spirometric restrictive pattern; TyG, triglyceride-glucose index; VIF, variance inflation factor.

LLN values were 81%, 82%, and 73% for FEV1%, FVC%, and FEV1(L)/FVC (L) ratio (%), respectively.

**.**
